# Anti-Obesity Effect and Signaling Mechanism of Potassium Poly-γ-Glutamate Produced by *Bacillus subtilis Chungkookjang* in High-Fat Diet-Induced Obese Mice

**DOI:** 10.3390/nu16060809

**Published:** 2024-03-12

**Authors:** Seung-Hyeon Lee, Jiwon Choi, Jae Young Park, Ha-Rim Kim, Myeongkuk Shim, Kyunghyun Im, Hyeonjeong Choe, Jae-Chul Choi, Young-Chul Park, Tae-Gyu Lim, Hyangyim Seo, Hansu Jang, Boung-Jun Oh, Seon-Young Kim, Mi Hee Park

**Affiliations:** 1 Jeonju AgroBio-Materials Institute, Wonjangdong-gil 111-27, Deokjin-gu, Jeonju-si 54810, Republic of Korea; sh94@jami.re.kr (S.-H.L.); jjay1205@jami.re.kr (J.Y.P.); poshrim@jami.re.kr (H.-R.K.); bjohkim@jami.re.kr (B.-J.O.); 2 KD Healthbio Co., Ltd., Suwon-si 16226, Republic of Korea; jw024321@kdhealthbio.com (J.C.); qisi1212@kdhealthbio.com (M.S.); icarrus@kdhealthbio.com (K.I.); choehjung@kdhealthbio.com (H.C.); 3 BL Corporation, Yongin-si 16827, Republic of Korea; cjc1151@bioleaders.com (J.-C.C.); andy@bioleaders.com (Y.-C.P.); 4 Department of Food Science & Biotechnology, Sejong University, Seoul 05006, Republic of Korea; tglim@sejong.ac.kr; 5 Jeonbuk Institute for Food-Bioindustry, Wonjangdong-gil 111-18, Deokjin-gu, Jeonju-si 54810, Republic of Korea; hiseo@jif.re.kr (H.S.); jhs@jif.re.kr (H.J.)

**Keywords:** potassium poly-γ-glutamate, *Chungkookjang*, obesity, inflammation, insulin resistance

## Abstract

The purpose of this work was to examine the effects of potassium poly-γ-glutamate (PGA-K) on mice fed a high-fat diet consisting of 60% of total calories for 12 weeks. PGA-K administration reduced the increase in body weight, epididymal fat, and liver weight caused by a high-fat diet compared to the obese group. The triglyceride, low-density lipoprotein cholesterol and high-density lipoprotein cholesterol levels, which are blood lipid indicators, were significantly increased in the obese group but were significantly decreased in the PGA-K-treated group. The administration of PGA-K resulted in a significant inhibition of pro-inflammatory cytokines, including tumor necrosis factor α and interleukin 6. Moreover, the levels of leptin and insulin, which are insulin resistance indicators, significantly increased in the obese group but were significantly decreased in the PGA-K-treated group. These results suggest that PGA-K exhibits a protective effect against obesity induced by a high-fat diet, underscoring its potential as a candidate for obesity treatment.

## 1. Introduction

Obesity is recognized as a worldwide epidemic and is linked to a spectrum of health issues, such as type 2 diabetes mellitus, hypertension, dyslipidemia, cardiovascular diseases, gallbladder disease, stroke, myocardial infarction, fatty liver disease, osteoarthritis, gout, mental disorders, and specific types of cancers [1,2]. Obesity is characterized by the conversion of excess energy from increased food intake into triglycerides (TGs) which are subsequently stored in adipose tissue, resulting in weight gain [3].

Obesity is associated with the expansion of fat cells (adipocytes) and inflammation in the surrounding adipose tissues [4]. Persistent lipid buildup in adipose tissue is the source of inflammation associated with obesity [5]. Insulin resistance is actively promoted by the release of inflammatory cytokines from adipose tissue, which also increases the risk of metabolic diseases associated with obesity [6].

Several studies have suggested that natural products sourced from edible and medicinal plants have anti-obesity effects with minimal to no side effects [7]. Various studies have been undertaken to hinder the accumulation of lipids and the synthesis of pro-inflammatory cytokines using various food materials [8]. Extracts of functional foods often contain components including polyphenols and flavonoids that have been shown to have the ability to reduce inflammation and cholesterol buildup associated with metabolic disorders including hypertension and obesity [9]. In situations of excessive fat accumulation, dysfunctional expanded adipocytes release different pro-inflammatory adipokines, including interleukin (IL) 6 and IL-1β and tumor necrosis factor (TNF) α [10]. The expansion of adipocytes also increases adipocyte death and recruitment of macrophages to adipose tissue [11]. Many crown-like structures are created during this adipocyte remodeling process, which is characterized by macrophages around dead adipocytes [12]. An increased prevalence of crown-like structures is associated with inflammation in adipose tissue [13].

Fermented soybean-based foods contain a diverse array of bioactive compounds that have potential therapeutic effects, particularly for metabolic disorders [14]. *Chungkookjang* is made by organically introducing *Bacillus subtilis* into the fermentation process by fermenting boiling soybeans with rice straw [15]. During the fermentation process of *chungkookjang*, soy protein is broken down into amino acids by the activity of a powerful proteolytic enzyme synthesized by B. subtilis. This not only enhances digestibility but also contributes to an increase in the vitamin B2 and calcium content of the final product [16]. The primary component found in the viscous mucous substance produced during fermentation is poly-γ-glutamic acid (γ-PGA), along with modified isoflavone compounds. PGA is recognized for its health benefits as it contributes to the absorption of calcium in the body [17,18]. Isoflavones, the main bioactive components of soybeans, play a critical role in enhancing the absorption and bioavailability of nutrients from fermented soybeans [19]. When *chungkookjang* is fermented, different physiologically active chemicals and enzymes that are absent from raw soybeans are produced [20]. These components play a role in preventing atherosclerosis, heart disease and inflammation mediated by oxidative stress, obesity, diabetes, senile dementia, cancer, and osteoporosis [15,21]. Moreover, these components exhibit various beneficial activities including lipid-lowering, blood pressure-lowering, thrombolytic, anti-mutagenic, immunostimulatory, anti-asthmatic, anti-androgenetic, antibacterial anti-alopecia effects, alongside attributes that promote skin enhancement [22]. This study seeks to investigate the impact of γ-PGA potassium salt (PGA-K) produced by *B. subtilis chungkookjang* on obesity associated with inflammation induced by a high-fat diet (HFD) mouse model and to assess the potential of PGA-K for obesity treatment.

## 2. Materials and Methods

### 2.1. Preparation of PGA-K

PGA-K was prepared using *B. subtilis chungkookjang*. *B. subtilis chungkookjang* (KCTC 0697BP) was inoculated into a preparative basic medium [GS basic medium with 5% l-glutamic acid, 5% glucose, 1%, (NH_4_)_2_SO_4_, 0.27% KH_2_PO_4_, 0.42% Na_2_HPO_4_·12H_2_O, 0.05% NaCl 0.05%, pH 6.8] and cultured at a 150 rpm stirring rate, an aeration rate of 1 vvm, and 37 °C for 36 h. Then, 2N hydrochloric acid was added to the solution and was let stand at 10 °C for 12 h to obtain a γ-PGA precipitate. After 12 h, the solution was filtered through a Nutsche filter, and the filtered PGA precipitate was thoroughly washed with distilled water. γ-PGA has a molecular mass of 1–15,000 kDa, and separate experiments were performed on subfractions with different molecular masses. To obtain γ-PGA potassium salt, γ-PGA was solubilized in 5 N KOH. The molecular mass of PGA-K was determined by gel permeation chromatography. Briefly, the PGA-K solution was diluted with 0.1 M NaNO_3_ and injected into the gel permeation chromatograph equipped with a ViscoGel GMPWXL xl column (7.8 mm × 30 cm; Viscotek, Houston, TX, USA), which was equilibrated with 0.1 M NaNO_3_ at 40 °C with a flow rate of 0.8 mL/min, and a Viscotek LR25 laser refractometer (Viscotek).

### 2.2. Animals

Male C57BL/6 mice of a particular pathogen-free grade, aged four weeks, were acquired from Damul Science (Daejeon, Republic of Korea) and were housed for one week. In a mouse cage with a controlled 12 h light/dark cycle, 22 °C ± 2 °C temperature and 55% ± 5% relative humidity, the mice were kept during the study. Prior to the start of the investigation, all experimental protocols were approved by the Animal Care Committee of the Jeonju AgroBio-Materials Institute, Jeonju, Republic of Korea (permission number: JAMI IACUC 2023003).

### 2.3. Experimental Groups

The mice were divided into five groups, the normal group (N), the high-fat diet-induced obesity group (HFD), the positive control group (PC), and the PGA-K administration group (PGA-K), each consisting of 7~8 mice. To induce obesity, the HFD, PC, and PGA-K groups were fed a 60% kcal fat diet for 12 weeks, and the N group was fed a normal diet (10% kcal fat). The PC and PGA-K groups were orally administered garcinia (300 mg/kg) and PGA-K (100 mg/kg and 200 mg/kg), respectively, for 12 weeks. N and HFD groups were administered vehicle (distilled water). The N and PC groups consisted of 7 mice per group, and the HFD and PGA-K groups consisted of 8 mice per group.

### 2.4. Evaluation of Biomarkers in Serum

Using the ELISA kit from R&D Systems (Abingdon, UK), the amounts of TNF-α, IL-6, and leptin in serum were determined. Concentrations of triglyceride (TG), total cholesterol (TC) and high-density lipoprotein (HDL) were determined using the kit provided by Asan Pharm (Seoul, Republic of Korea), and low-density lipoprotein (LDL) and insulin levels were measured using kits provided by CrystalChem (Elk Grove, CA, USA). The manufacturer’s instructions were followed for all the measurements.

### 2.5. Histology

Mice liver and adipose tissues were embedded in paraffin after being treated in 4% paraformaldehyde. Hematoxylin and eosin (H&E) were used for adipose tissues and Oil Red O for liver tissues to stain tissue sections at a thickness of 4 μm.

### 2.6. Statistical Analyses

With Sigmaplot v16.0 (Systat Software Inc., San Jose, CA, USA), all statistical analyses were carried out, and the results are shown as means ± standard deviation. After conducting a one way analysis of variance and Duncan’s multiple comparison test, statistical analysis was used to find differences. To evaluate differences between three or more groups on all observed parameters, these analyses were used. *p* < 0.05 was used to indicate statistical significance. To evaluate differences of means, we used a *t*-test and revealed means ± standard error of mean (SEM), 95% CI and *p* value.

## 3. Results

### 3.1. Effects of PGA-K Administration on Body Weight and Food Intake in HFD-Fed Mice

**Figure 1:**
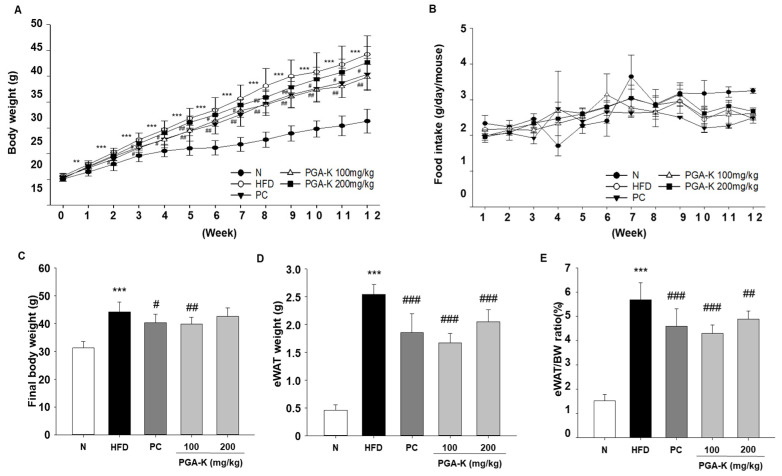
Effects of PGA-K on body weight and eWAT weight in HFD-induced obesity mice. (**A**) PGA-K effects on body weight gain, (**B**) food intake, (**C**) final body weight, (**D**) eWAT weight, and (**E**) the ratio (%) of eWAT to body weight for 12 weeks of obesity induction by HFD. The mean ± SD is represented by each value. For data analysis, Duncan’s multiple comparison test was used. ** *p* < 0.01 and *** *p* < 0.001, versus the N group; ^#^ *p* < 0.05, ^##^ *p* < 0.01, and ^###^ *p* < 0.001, versus the HFD group. PGA-K, poly-γ-glutamate potassium; HFD, high-fat diet; eWAT, epididymal white adipose tissue; N, normal; PC, positive control.

Initial body weight did not show any notable variances among the groups. From the first week of the experiment, the HFD group exhibited a notable rise in body weight compared to the N group. After 12 weeks, the final body weight of the HFD group showed a significant increase to 44.26 ± 3.6 g, which is about 1.4 times that of the N group, while the PGA-K administration group had a relatively low increase in body weight, and the 100 mg/kg PGA-K group showed a statistically significant effect (Figure 1A,C). Also, changes in body weight from baseline were analyzed between the PGA-K 100 mg/kg group and the HFD group (Table 1). The body weight changes were significantly decreased in the PGA-K 100 mg/kg administered group compared to the HFD group.

Epididymal white adipose tissue (eWAT) weight exhibited a significant increase in the HFD group compared to the N group following a 12-week period, but the PGA-K group showed a decrease in eWAT weight (Figure 1D). Moreover, the eWAT/body weight ratio was decreased by treatment with 100 and 200 mg/kg PGA-K (Figure 1E). Over the course of the investigation, no significant variations in food consumption were noted between the various experimental groups (Figure 1B). Hence, it can be inferred that PGA-K has therapeutic potential for HFD-induced obesity.

### 3.2. PGA-K Prevents Adipogenesis and Lipid Accumulation in HFD-Fed Mice

**Figure 2:**
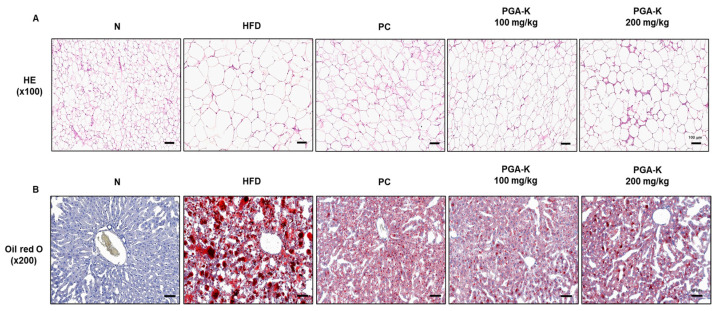
Histopathological assessment of the effects of PGA-K on the liver and eWAT of HFD-induced obesity mice. (**A**) Representative image of eWAT tissue stained with H&E. Magnification, 100×; scale bar, 100 μm. (**B**) Representative image of liver tissue stained with Oil Red O. Magnification, 200×; scale bar, 60 μm. PGA-K, poly-γ-glutamate potassium; HFD, high-fat diet; eWAT, epididymal white adipose tissue; PC, positive control; N, normal; H&E, hematoxylin and eosin.

For histological analysis, the eWAT tissue was stained with H&E. In the epididymal fat of obese mice, adipocytes were abnormally enlarged, and some adipocytes were damaged (Figure 2A). In the group administered with doses of 100 and 200 mg/kg of PGA-K, adipocyte heterogeneity and hypertrophy were reduced compared to the HFD group (Figure 2A). To evaluate whether PGA-K inhibits HFD-induced hepatic steatosis, histological analysis was performed on Oil Red O-stained liver sections. The liver tissue of the HFD group showed numerous red lipid droplets with incomplete cellular structure and fat accumulation, which suggested that substantial accumulation of lipid occurred in the liver tissue (Figure 2B). On the other hand, both groups administered 100 and 200 mg/kg of PGA-K showed decreased cell deformation and lipid accumulation in liver tissue (Figure 2B). These results showed that PGA-K had a preventive effect on adipocyte hypertrophy and intrahepatic lipid accumulation in HFD-induced obese mice.

### 3.3. Effects of PGA-K on Serum Lipid Profiles in HFD-Fed Mice

**Figure 3:**
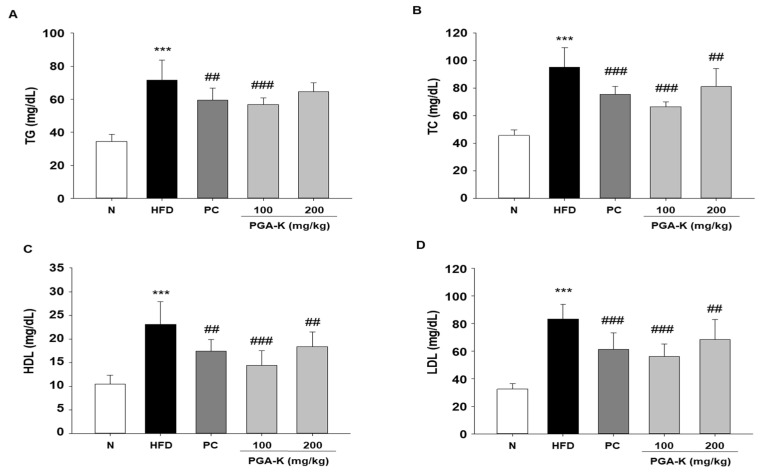
Effects of PGA-K on serum lipid profiles of HFD-induced obesity mice. The levels of (**A**) TG, (**B**) TC, (**C**) HDL, and (**D**) LDL. All values represent the mean ± SD. Duncan’s multiple comparison test was utilized for data analysis. *** *p* < 0.001, versus the N group; ^##^ *p* < 0.01 and ^###^ *p* < 0.001, versus the HFD groups. PGA-K, poly-γ-glutamate potassium; HFD, high-fat diet; TG, triglyceride; TC, total cholesterol; HDL, high-density lipoprotein; LDL, low-density lipoprotein; N, normal; PC, positive control.

The serum TG, TC, HDL, and LDL levels of mice in each group were measured using ELISA. The levels in the HFD group were substantially higher than those in the N group. After PGA-K administration for 12 weeks, the levels of TG (Figure 3A), TC (Figure 3B), HDL (Figure 3C), and LDL (Figure 3D) decreased.

### 3.4. Effects of PGA-K on Liver Damage in HFD-Fed Mice

**Figure 4:**
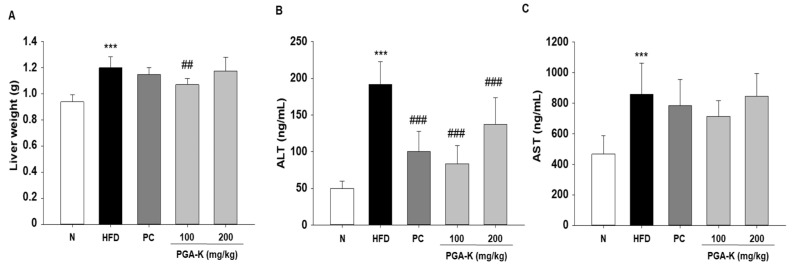
Effects of PGA-K on liver toxicity in HFD-induced obesity mice. (**A**) Liver weight and (**B**) serum ALT and (**C**) AST levels in HFD-induced obesity mice. All values are presented as the mean ± SD. Duncan’s multiple comparison test was utilized for data analysis. *** *p* < 0.001, versus the N group; ^##^ *p* < 0.01 and ^###^ *p* < 0.001, versus the HFD group. PGA-K, potassium poly-γ-glutamate; HFD, high-fat diet; ALT, alanine aminotransferase; AST, aspartate aminotransferase; N, normal; PC, positive control.

To test whether PGA-K inhibits liver damage, along with serum, levels of aspartate aminotransferase (AST) and alanine aminotransferase (ALT) were tested. HFD-induced obese mice had significantly increased liver weight and serum ALT and AST levels. Liver weight was decreased in the PGA-K 100 mg/kg group (Figure 4A), and serum ALT was significantly decreased in both PGA-K 100 and 200 mg/kg groups (Figure 4B). In the case of serum AST levels, the average value seemed to decrease only in the PGA-K 100 mg/kg group. Nevertheless, no notable distinction was noted between the HFD and PGA-K groups (Figure 4C).

### 3.5. Effects of PGA-K on HFD-Induced Pro-Inflammatory Cytokines

**Figure 5:**
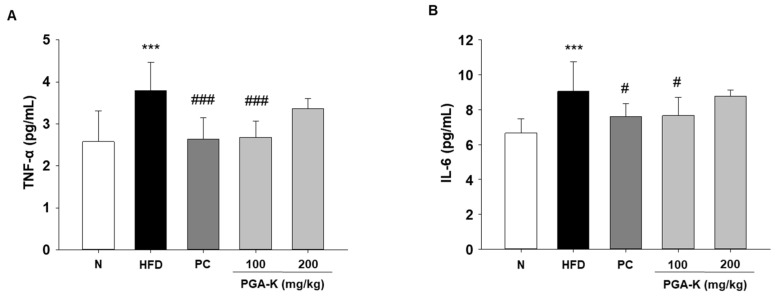
Effects of PGA-K on serum pro-inflammatory cytokine levels in HFD-induced obesity mice. The levels of (**A**) TNF-*α* and (**B**) IL-6 in HFD-induced obese mice serum. All values represent the mean ± SD. Duncan’s multiple comparison test was utilized for data analysis. *** *p* < 0.001, versus the N group; ^#^ *p* < 0.05, and ^###^ *p* < 0.001, versus the HFD group. PGA-K, poly-γ-glutamate potassium; HFD, high-fat diet; TNF-*α*, tumor necrosis factor *α*; IL-6, interleukin; N, normal; PC, positive control.

In order to comprehend the mechanisms behind obesity-induced lipid metabolism and inflammatory responses, we assessed the levels of inflammatory cytokines. In obesity, persistent inflammation is distinguished by aberrant expression of genes responsible for encoding pro-inflammatory cytokines [23]. Elevated levels of pro-inflammatory cytokines, such as TNF-α and IL-6, occur concomitantly with an increase in lipid content in white adipose tissue (WAT), contributing to the development of complications associated with obesity [24]. In this study, TNF-α and IL-6 levels were measured in serum using ELISA. The levels of serum TNF-α and IL-6 were notably elevated in the HFD group compared to the normal diet group (N). After administration of PGA-K for 12 weeks, TNF-α (Figure 5A) and IL-6 production (Figure 5B) decreased.

### 3.6. Effects of PGA-K on HFD-Induced Insulin Resistance

**Figure 6:**
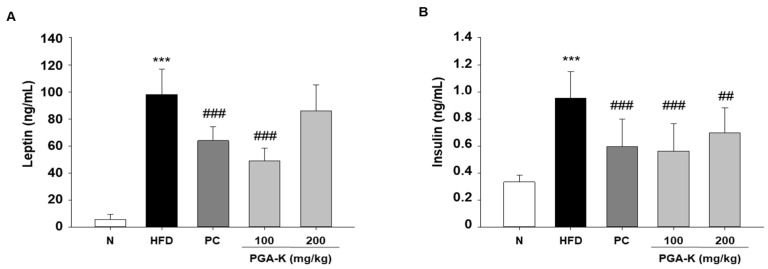
Effects of PGA-K on serum insulin resistance markers of HFD-induced obesity mice. (**A**) Leptin and (**B**) insulin. All values are presented as the mean ± SD. Data analysis was performed using Duncan’s multiple comparison test. *** *p* < 0.001, versus the N group; ^##^ *p* < 0.01 and ^###^ *p* < 0.001, versus the HFD group. PGA-K, poly-γ-glutamate potassium; HFD, high-fat diet; TNF-α, tumor necrosis factor α; IL-6, interleukin; N, normal; PC, positive control.

The secretion of pro-inflammatory molecules by adipose tissue actively contributes to the development of insulin resistance and increases the vulnerability to metabolic diseases associated with obesity [25]. Obesity-induced systemic low-grade inflammation heightens the risk of developing type 2 diabetes mellitus [26]. In addition, insulin resistance in overweight people often correlates with hyperinsulinemia, which is associated with obesity, dyslipidemia, and glucose intolerance [27]. To examine whether PGA-K is effective against insulin resistance, we determined the levels of leptin and insulin in serum using ELISA. HFD significantly increased leptin and insulin levels. After administration of PGA-K for 12 weeks, the production of leptin (Figure 6A) and insulin (Figure 6B) decreased.

## 4. Discussion

Excessive body fat accumulation delineates the hallmark of obesity. Inflammation induced by obesity arises from the accumulation of lipids in adipose tissue [28]. This inflammatory process is marked by an increase in both the number (hyperplasia) and size (hypertrophy) of adipocytes, leading to the enlargement of adipose tissue mass [29]. Adipose tissue acts as a natural calorie depot, expanding when there is an excess of nutrients, storing lipids, and releasing them during energy deficits [30]. Nevertheless, excessive fat accumulation and dysfunction in adipocytes result in changes in plasma lipid and lipoprotein levels. This includes an increase in LDL cholesterol levels and TG, along with a decrease in HDL cholesterol levels, contributing to the development of obesity-related disorders [31].

This study provides proof that PGA-K has anti-obesity effects in mice given a high-fat diet (HFD), with an emphasis on adipose tissue malfunction. According to the results, PGA-K significantly reduced the body weight that the HFD caused, especially fat tissue hypertrophy. PGA-K administration to HFD-induced obese mice significantly reduced relative liver size and fat pad size. The fat size in obese mice induced by HFD, however, treated with PGA-K decreased to match that of mice fed a normal-fat diet. Despite no notable impacts of PGA-K on food intake, mice treated with PGA-K exhibited lower body weight gain and white adipose fat mass compared to untreated mice. These findings suggest that alterations in caloric intake do not necessarily correspond to changes in adipose tissue mass. Moreover, PGA-K mitigated adipose tissue disorders induced by HFD, such as heightened inflammation, hinting at its capacity to alleviate metabolic issues associated with obesity. Moreover, PGA-K reduced the levels of insulin resistance markers, including insulin and leptin. Thus, *B. subtilis chungkookjang*-derived PGA-K is a potential candidate for reducing and managing obesity and obesity-related diseases.

The field of natural product research is continuously expanding, with ongoing efforts aimed at enhancing the biological activities of these compounds to address diverse diseases through treatment and prevention. Despite significant research efforts into the pathogenesis of obesity and ongoing exploration of novel treatment strategies, a definitive cure remains elusive. Treatment medications for obesity frequently have potential side effects and complications. Several anti-obesity medications, including liraglutide, naltrexone–bupropion, orlistat, and phentermine–topiramate have been approved by the US Food and Drug Administration (FDA) [32]. Meanwhile, numerous stimulant-type weight loss medications, such as phentermine and diethylpropion, are typically recommended for short-term use because of the risk of dependence and other potential side effects [33]. Certain drugs used in the clinical treatment of obesity are associated with adverse effects, including nausea, insomnia, constipation, gastrointestinal problems, and potential cardiovascular complications [34]. Therefore, numerous initiatives are underway to explore and cultivate anti-obesity foods and food ingredients that can effectively reduce body fat accumulation, decrease the risk of obesity-related chronic diseases, and reduce potential side effects associated with clinical treatments [35].

Fermented soybean products have been studied for their potential anti-obesity effects. These effects may be attributed to various bioactive compounds present in fermented soybeans, such as isoflavones, peptides, and polyphenols, which have been shown to modulate lipid metabolism, adipocyte differentiation, and inflammation. Additionally, the fermentation process may enhance the bioavailability and efficacy of these compounds. Research suggests that regular consumption of fermented soybean foods may contribute to weight management and the prevention of obesity-related metabolic disorders.

Fermented soybean products contain a variety of bioactive ingredients that may contribute to their anti-obesity effects. Soybean fermentation can produce bioactive peptides that have a variety of physiological benefits, including the ability to prevent obesity. These peptides may function as inhibitors of pancreatic lipase, an enzyme involved in the digestion and absorption of lipids, resulting in decreased absorption of fat [20,36]. Additionally, they might influence hormones that control hunger and increase fullness, which would lower calorie consumption. Fermented soybeans contain phytoestrogens called isoflavones, like genistein and daidzein. It has been demonstrated that they influence lipid metabolism through controlling adipocyte development, accelerating fat oxidation, and preventing fat from accumulating [37]. Furthermore, eating isoflavones has been linked to a decrease in triglycerides, LDL, and total cholesterol as well as an increase in HDL cholesterol [38]. Furthermore, soluble and insoluble dietary fibers are abundant in fermented soybean products. Dietary fiber slows down the emptying of the stomach and increases sensations of fullness, which helps to promote satiety and control hunger [39]. Additionally, fiber can modulate gut microbiota composition, leading to improved metabolic health and reduced obesity risk. Fermented soybean products often contain probiotic bacteria, such as Lactobacillus and Bifidobacterium species, which can influence gut microbiota composition and function [40]. Probiotics may improve insulin sensitivity, decrease fat absorption, and increase energy expenditure—all of which are advantageous for controlling weight and preventing obesity [41]. Research from clinical and experimental settings shows that being overweight or obese increases the risk of type 2 diabetes mellitus, with obesity significantly raising the risk. Furthermore, even with strict management of blood pressure, cholesterol, and glucose, those with type 2 diabetes mellitus have a higher risk of cardiovascular disease [42]. Additionally, a number of studies have connected these outcomes to the bioactive elements of fermented soybean meals, including protease inhibitors, hemagglutinin, and alpha-amylase and alpha-glucosidase inhibitors. These substances may disrupt regular metabolism and help treat obesity and other metabolic disorders [14]. Antioxidants with anti-inflammatory qualities, such as flavonoids and polyphenols, are found in fermented soybean products. Antioxidants may help reduce inflammation and its detrimental effects on metabolic health since chronic low-grade inflammation is linked to obesity and metabolic syndrome [9]. Overall, these bioactive components of fermented soybean products may work together to modulate lipid metabolism, appetite regulation, the composition of the gut microbiota, and inflammation, among other pathways, to produce their anti-obesity effects.

Moreover, in a previous investigation, it was documented that soy isoflavones markedly attenuate body weight gain and fat accumulation in both obese and lean rats [43]. Moreover, a study report suggested that supplementation with *chungkookjang* enhances lean body mass while diminishing visceral fat areas. Another study also suggested that a diet rich in isoflavones improves lipid metabolism and exerts anti-obesity effects [44]. Daidzein and genistein, found abundantly in fermented soybean foods, are acknowledged for their bioactive properties, influencing lipid metabolism and thermogenesis in vivo [14,45]. Aglycones and metabolites demonstrate their anti-obesity effects by affecting processes such as lipogenesis (the synthesis of fatty acids and triglycerides), hyperlipidemia (elevated levels of fat particles in the blood), hyperglycemia (increased blood glucose levels), and enhanced insulin resistance [46]. γ-PGA is a hydrolyzed biopolymer composed of L-glutamic acid and/or D-glutamic acid monomers produced when *Bacillus subtilis* ferments soybeans [17,18,47]. Studies have reported that γ-PGA has various physiological activities, including potential anti-obesity and anti-inflammatory properties [48,49]. Preliminary research has demonstrated the anti-obesity qualities of isoflavones and soy proteins. Similar results were shown in this trial with PGA-K therapy. Additionally, the process of adipocyte differentiation is paralleled by an upregulation in the expression of inflammatory cytokines [10]. Thus, we tested the inflammatory cytokine expression in WATs, and the results showed that PGA-K treatment decreased the expression of TNF-*α* and IL-6 in serum. Adipose tissue releases adipocytokines, including TNF-α, IL-6, leptin, and adiponectin, and elevated serum leptin and reduced serum adiponectin levels are characteristic features of obesity [50]. In another study, prolonged consumption of an HFD resulted in heightened levels of insulin in the serum, triggered insulin resistance, and promoted increased fat accumulation within the liver [51]. In the present study, we found that PGA-K decreased serum insulin and leptin levels, indicating its potential in improving insulin clearance and significantly reducing fat accumulation.

Our research findings provide compelling evidence for the beneficial effects of PGA-K administration on obesity. Through comprehensive analysis, we observed significant improvements in various parameters associated with obesity, including body weight, adipose tissue weight, and histological features. Remarkably, administration of PGA-K resulted in a significant reduction in both body weight and adipose tissue weight, suggesting its potential as an effective intervention for obesity management. Moreover, our study revealed promising outcomes related to metabolic health, as PGA-K administration was associated with the restoration of insulin and leptin production. We summarized the results of various parameters in Table 2. These findings suggest that PGA-K may play a crucial role in preventing the onset or progression of diabetes mellitus, a common comorbidity of obesity. In summary, our results strongly support the notion that PGA-K exerts a preventive effect against obesity and its associated metabolic complications. Further research is warranted to elucidate the underlying mechanisms and optimize the therapeutic potential of PGA-K for combating obesity and related health conditions.

## 5. Conclusions

This research offers compelling scientific evidence supporting the anti-obesity properties of PGA-K in mice fed a high-fat diet, focusing on the dysfunction of adipose tissue. The study revealed that PGA-K, derived from *B. subtilis chungkookjang* significantly reduced body weight gain induced by the high-fat diet, particularly adipose tissue hypertrophy. Moreover, PGA-K mitigated obesity-related adipose tissue disorders induced by the high-fat diet, including inflammation, suggesting its potential to alleviate metabolic disorders associated with obesity. Hence, PGA-K emerges as a promising candidate ingredient in functional foods for the reduction and management of obesity and obesity-related diseases.

## Figures and Tables

**Table 1:** Change in body weight from baseline.

Time Laps (Weeks)	PGA-K 100 mg/kg	HFD	Difference in Means (SE; 95%CI)	*p* Value
Baseline	20.26 ± 0.53	20.66 ± 0.43		
1	2.17 ± 0.23	2.25 ± 0.27	−0.08 (0.36, −0.84 to 0.68)	0.82
2	4.20 ± 0.32	4.55 ± 0.36	−0.36 (0.48, −1.93 to 0.68)	0.47
3	6.02 ± 0.42	7.05 ± 0.52	−1.03 (0.66, −2.46 to 0.40	0.14
4	7.46 ± 0.37	8.99 ± 0.65	−1.53 (0.75, −3.14 to 0.08)	0.06
5	9.2 ± 0.43	11.2 ± 0.72	−1.20 (0.84, −3.8 to −0.19)	0.03
6	11.14 ± 0.44	12.8 ± 0.90	−1.66 (1.0, −3.81 to 0.49)	0.12
7	13.08 ± 0.51	14.99 ± 0.97	−1.91 (1.10, −4.26 to 0.45)	0.10
8	14.24 ± 0.62	17.48 ± 1.18	−3.24 (1.33, −6.1 to −0.38)	0.03
9	15.78 ± 0.89	19.34 ± 1.11	−3.57 (−6.62, 0.51 to −0.51)	0.03
10	17.16 ± 0.96	20.19 ± 1.27	−3.03 (1.59, −6.44 to 0.38)	0.08
11	17.79 ± 0.94	21.62 ± 1.21	−3.84 (1.54, −7.13 to −0.54)	0.03
12	19.60 ± 1.02	23.60 ± 1.20	−4.00 (1.58, −7.39 to −0.60)	0.02

**Table 2:** Summary of efficacy parameters.

Efficacy Parameters	PGA-K 100 mg/kg	HFD	Difference in Means	*p* Value
TG	56.88 ± 1.43	71.61 ± 4.30	−14.74	<0.001
TC	66.52 ± 1.30	95.31 ± 5.02	−28.79	<0.001
HDL	14.41 ± 1.11	23.12 ± 1.70	−8.71	<0.001
LDL	56.21 ± 3.17	83.44 ± 3.69	−27.23	<0.001
eWAT weight	1.67 ± 0.60	2.54 ± 0.06	−0.87	<0.001
eWAT/BW (%)	4.29 ± 0.12	5.68 ± 0.25	−1.39	<0.001
Liver weight	1.07 ± 0.02	1.20 ± 0.03	−0.13	0.002
ALT	83.49 ± 8.84	191.83 ± 11	−108.34	<0.001
AST	714.69 ± 36.40	860.42 ± 71.86	−145.73	0.09
TNF-a	2.68 ± 0.14	3.80 ± 0.24	−1.12	<0.001
IL-6	7.67 ± 0.37	9.05 ± 0.60	−1.38	0.017
Leptin	49.08 ± 3.22	97.99 ± 6.70	−48.91	<0.001
Insulin	0.56 ± 0.07	0.96 ± 0.07	−0.39	<0.001

## Data Availability

The data presented in this study are available in this article.
